# The Behaviour of Both *Listeria monocytogenes* and Rat Ciliated Ependymal Cells Is Altered during Their Co-Culture

**DOI:** 10.1371/journal.pone.0010450

**Published:** 2010-05-04

**Authors:** Mina J. Fadaee-Shohada, Robert A. Hirst, Andrew Rutman, Ian S. Roberts, Chris O'Callaghan, Peter W. Andrew

**Affiliations:** 1 Department of Infection, Immunity and Inflammation, University of Leicester, Leicester, United Kingdom; 2 Faculty of Life Sciences, University of Manchester, Manchester, United Kingdom; Columbia University, United States of America

## Abstract

**Background:**

Ciliated ependymal cells line the cerebral ventricles and aqueducts separating the infected CSF from the brain parenchyma in meningitis.

**Principal Findings:**

Investigation of the interaction of *Listeria monocytogenes* with cultured rat brain ependymal cells showed that certain strains reduced the beat frequency of the cilia but all the strains studied significantly reduced the ciliary beat amplitude (the linear distance travelled by the tip of each cilium per beat cycle).

**Conclusion:**

The presence of the ependyma caused aggregation of some listeria strains and in some cases extracellular material also was seen in association with bacterial aggregates. These observations were dependent on the expression of genes required for invasion, intracellular survival and listerial cell to cell spread that are regulated by the transcriptional activator, positive regulatory factor A (PrfA).

## Introduction

*Listeria monocytogenes* is a food-borne pathogen that, if ingested, has the potential to enter the systemic circulation and move from the blood to penetrate the blood-brain barrier to cause meningitis and meningoencephalitis, especially in the unborn and newborn infant [1], [2]. The risk of listeriosis is also markedly increased in immunocompromised patients, such as those with AIDS [3].

In meningitis, bacteria in the cerebrospinal fluid are separated from the neuronal tissue adjacent to the ventricular system and aqueducts, by the ependyma. The ependyma is a single, uninterrupted, layer of ciliated cells that lines the cerebral ventricles, cerebral aqueducts and the central canal of the spinal cord. Each ependymal cell has approximately 40 cilia that beat continuously at a frequency of around 40 Hz, moving the CSF close to the ventricular wall, in the direction of CSF flow [4]. In addition, the ependymal cilia are thought to act as a barrier to pathogen infection of the underlying neuronal tissue [5]. Prior to the entry of listeria into the cells of the brain the bacteria need to overcome this mechanical beating of the cilia. Abnormal movement of the ependymal cilia has been strongly linked to the development of hydrocephalus [6], [7], [8].

During meningitis caused by *L. monocytogenes*, bacteria have been shown to be present inside ependymal [9] and neuronal [10] cells, as well as in the cerebrospinal fluid [9], [11], [12]. However, the details of *L. monocytogenes* attachment to ciliated ependymal cells and subsequent invasion are poorly understood.

The expression of genes required for invasion, intracellular survival and listerial cell to cell spread is dependent upon a transcriptional activator known as PrfA (positive regulatory factor A) [13], [14], [15]. PrfA was first identified as a regulatory factor required for listeriolysin-O transcription and has since been shown to regulate the expression of a large number of bacterial gene products directly associated with virulence [16]. To date, only 10 of the 2,853 coding sequences of the *L. monocytogenes* EGDe genome [17] have been confirmed to be directly regulated by PrfA [18], [19].

It is thought that PrfA serves as a switch enabling *L. monocytogenes* transition from the outside environment into an animal host [20]. The absolute requirement of PrfA for *L. monocytogenes* virulence has been demonstrated, as has the requirement for several genes within the PrfA regulon [21], [22]. Strains with mutations within the PrfA gene failed to replicate within the cytosol of host cells or failed to spread into adjacent cells [15], [21]. These mutants also were severely attenuated for virulence in murine models of listeriosis [21].

## Materials and Methods

### Preparation of bacterial suspensions

*Listeria monocytogenes* strains used in this study are listed in Table 1. Listeria were grown in 200 ml tryptose soya broth overnight at 37°C with shaking at 200 rpm. The bacteria were then sedimented (4,000×g for 10 min) and re-suspended in 10 ml medium 199 (Invitrogen, UK) containing 10% v/v glycerol and stored at −70°C until required. Before use, frozen stocks were thawed at room temperature. Numbers of viable bacteria were determined by colony counting on tryptose soya broth agar plates. For growth of EGDe *ΔprfA* and 10403S *ΔprfA*, 5 mg/ml erythromycin was added to the medium. For use, listeria were diluted to 2×10^8^ cfu/ml in tissue culture medium 199.

**Table 1:** *Listeria monocytogenes* strains.

Strain	Description	Reference
10403S	*L. monocytogenes* wild-type strain	[22]
C52	*L. monocytogenes* wild-type strain	[40]
EGDe	*L. monocytogenes* wild-type strain	[41]
10403SΔ*prfA*	*prfA* gene disrupted by integration of *Tn* 917	[42]
EGDeΔ*prfA*	*prfA* gene disrupted by integration of pAUL51-10	[22]

### Ependymal cell culture

The research using new born rat brains was conducted according to national guidelines under the UK, Animals (Scientific Procedures) Act 1986 and all animals were born in the University of Leicester Biomedical Sciences Unit. The research on the ex-vivo tissue was approved by the University of Leicester ethics committee.

An adaptation of a previously described method [23] was used to grow the ependymal cells. Eight-well rectangular (25×35 mm) tissue culture trays (Fisher, UK) were coated with bovine fibronectin (35 µg/cm^2^) and were incubated at 37°C in 5% CO_2_ for 2 hours before use. Following cervical dislocation, the brains of newborn (1 to 2 day old) Wistar rats were removed. The cerebellum was removed, as were small (3 mm) edge regions of the frontal cortex and the left and right cortical hemispheres. The remaining brain regions (containing ependymal cells and ventricles) were mechanically dissociated in 2 ml of tissue culture medium 199 [24]. Dissociated tissue from each brain was seeded (500 µl/well) into the wells of 25×35 mm tissue culture trays; each well contained 2 ml of medium. The medium was serum-free minimum essential medium (Gibco Life Technologies, Paisley, UK) containing penicillin (100 IU/ml), streptomycin (100 µg/ml), fungizone (2.5 µg/ml), bovine serum albumin (5 µg/ml), insulin (5 µg/ml), transferrin (10 µg/ml), selenium (5 µg/ml) (Invitrogen, UK) and from day 3 onward, thrombin (0.5 IU/ml) (Sigma-Aldrich, UK) [24]. The medium was replaced on day 3 after seeding. Thereafter, the adherent ependymal cells were fed by the replacement of 2 ml of medium, three times a week [24]. The ependymal cells grew as colonies within the wells and the beating cilia were used to identify them at day 5. The ciliated cells were used for experiments when cells were around two weeks old [23]. The number of cells in each well varied between 1000–10000 cells. The experiments were done in 8.58 cm^2^ wells in 2 ml of media containing 1^8^ cfu/ml, and if we correct for volume above the cells for an average cell colony surface area (4800 cells, each 10 µm in diameter, ∼0.48 mm^2^×2.3 mm height of media above the colony  = 1.1 mm^3^). The total volume added was 12.6 mm^3^, therefore, the cells will be in contact with 2^8^ cfu/(12.6/1.1)/4800 approximately equal to 3654 bacteria per cell. Before use the MEM medium was completely removed from each well and the cells were washed 5 times with PBS and 1 ml of medium 199 was added to each well. For infection, 1 ml of *L. monocytogenes* suspension was added, to give a final concentration of 1×10^8^ cfu/ml.

### Measurement of ciliary beat frequency

Ciliary beat frequency was measured as previously described [24], [25]. Briefly, trays of ependymal cells were placed in a humidified (80 to 90% humidity) thermostatically controlled (37°C) incubation chamber on a light microscope stage (Diphot; Nikon). All measurements were taken with the solution temperature between 36.5 and 37.5°C and the pH between 7.35 and 7.45. Beating cilia were recorded (32x objective) using a troubleshooter 1000 high speed video camera (Lake Image Systems Ltd, UK) at 500 frames per second. All video files were created using the AVI video format. For viewing the AVI files MiDAS 4.0 player software (http://www.xcitex.com/html/downloads.php) was used. Video sequences were played back either at reduced frame rates or frame by frame and ciliary beat frequency was determined by timing a pre-selected number of individual ciliary beat cycles. At each time point, measurements of twenty individual cilia from the same colony of cells were made and averaged. This was repeated in 5–11 independent experiments (i.e. A total of 100–220 CBF readings were made).

### Measurement of ciliary beat amplitude

Cilia were viewed using an inverted microscope (Diphot; Nikon, UK). High-speed video recordings were made, as described above, to measure ciliary beat frequency. Slow motion playback of the high-speed video recordings enabled us to visualise the cilia tips. We were then able to determine the maximum forwards movement of the cilia and the maximum backwards movement of the cilia tips. The distance travelled between these two points was measured on the display screen and defined as the ciliary beat amplitude.

For each experiment, five different regions (62.5 µm×140 µm) of ciliated ependyma were chosen at random and from each area five measurements of ciliary beat amplitude were made. If listeria aggregates were seen, five readings were taken from cilia covered by these aggregates and five readings from cilia outside these areas. The amplitude of the ciliary beat in the control that was not exposed to listeria was defined as 100%.

### Measurement of bacterial aggregation

Still photographs were obtained from various stages of the experiment. Each photograph was equivalent to 62.5 µm×140 µm of the ciliated ependyma. From these photographs, the perimeter of *L. monocytogenes* aggregates was determined by image analysis. The percentage of ciliated tissue covered by listerial aggregate was determined for each sample.

### Scanning electron microscopy

Ependymal cells were fixed with 4% v/v glutaraldehyde in Sorensen's buffer (pH 7.4) [24] for 48 hours and then rinsed 3 times in Sorensen's buffer. The cells were post-fixed in 1% w/v osmium tetroxide for 1 hour and rinsed in Sorensen's buffer. The samples were then dehydrated through a graded ethanol series and immersed in hexamethyldisilazane (HMDS). The HMDS was allowed to evaporate, leaving the cells fixed to aluminium scanning electron microscope stubs. These were then sputter-coated with gold prior to examination [24].

### Statistical analysis

All data are expressed as the mean ± standard deviation of 5 to 11 independent experiments. Data were analysed by one way analysis of variance (ANOVA), and if significant (p<0.05), individual comparisons were made using an unpaired Students t-test. The Bonferroni correction test was used for repeated measurements.

## Results

### Interaction of wild type *L. monocytogenes* and ciliated ependymal cells

**Figure 1:**
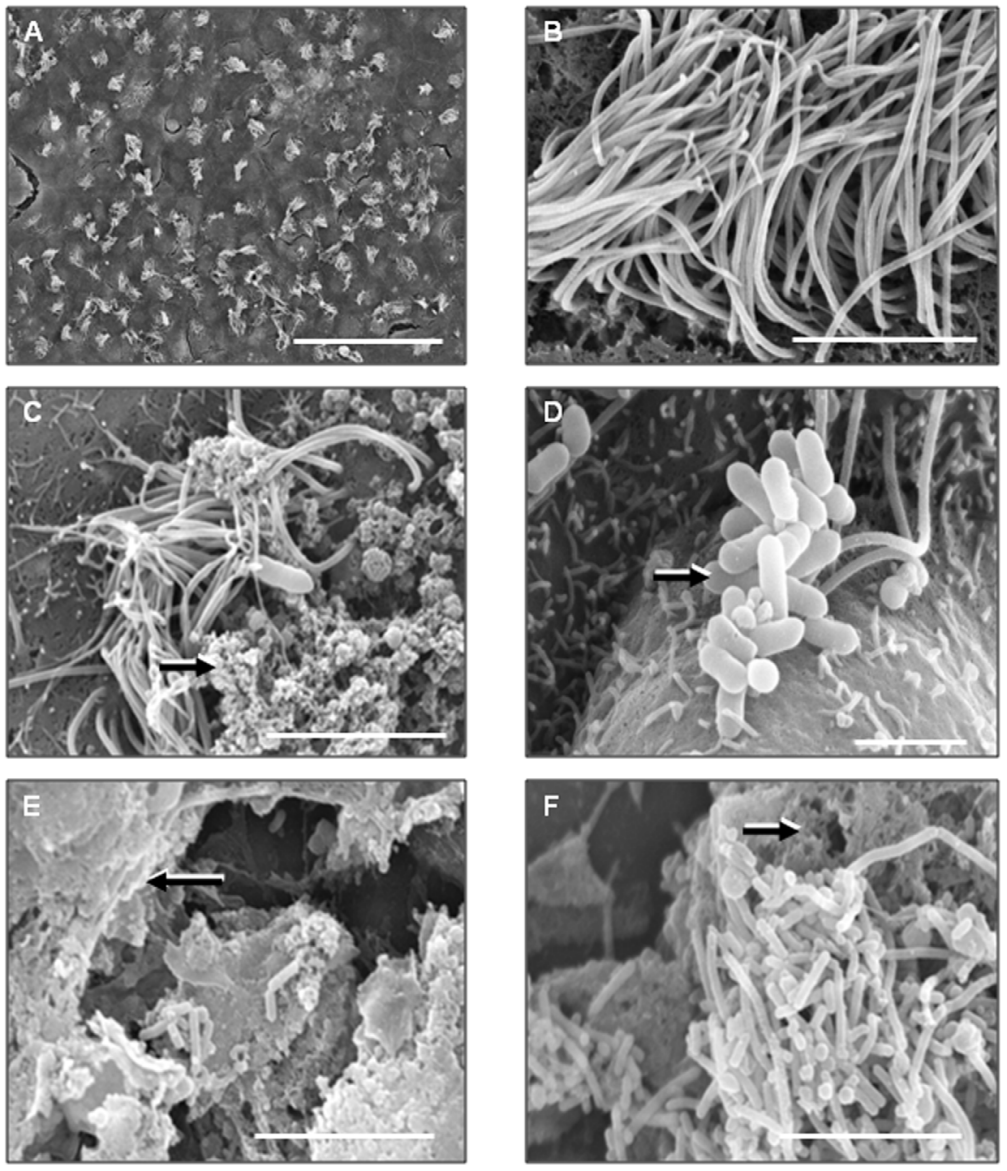
Scanning electron microscope images of Listeria on ciliated ependymal cells. (A) low magnification of rat ciliated ependymal cells *in vitro*. Scale bar represents 70 µm, (B) high magnification of a rat ciliated ependymal cell *in vitro*. Scale bar represents 5 µm, (C) *L. monocytogenes* strain 10403S on ciliated ependymal cell. The arrow shows areas of extracellular material. Scale bar represents 4 µm, (D) *L. monocytogenes* strain 10403S aggregate (arrow) on ciliated ependymal cells. No extracellular material is visible. Scale bar represents 2 µm, (E) presence of a thick layer of extracellular material on ciliated ependymal cells after incubation with *L. monocytogenes* strain EGDe. The arrow is pointing to an area with extracellular material. Scale bar represents 5 µm, (F) *L. monocytogenes* EGDe aggregate in the presence of extracellular material. The arrow is pointing to an area of extracellular material. Scale bar represents 5 µm.

Ependymal cells from newborn Wistar rats were grown in culture until ciliated (Fig. 1A, 1B and S1) and then were incubated with wild-type strains of *L. monocytogenes* for three hours at 37°C. The behaviour of both the ependymal cilia and the listeria were altered in these co-cultures but the alterations were dependent on the strain of *L. monocytogenes*.

The first strain studied was *L. monocytogenes* 10403S. In the presence of ependymal cells the bacteria aggregated and by three hours about 10% of the surface area of the ependymal cell culture was covered by listerial aggregates that were attached to the underlying cilia (Table 2). Scanning electron microscopy revealed that the vast majority of the 10403S aggregates consisted of bacteria within an extracellular material (Fig. 1C). However, occasional aggregates of bacteria associated with cilia but without extracellular material were also revealed (Fig. 1D).

**Table 2:** Ependymal ciliary beat frequency and percentage of tissue covered with bacterial aggregates in the presence of extracellular material.

*L. monocytogenes* strain	ciliary beat frequency (Hz)	% of tissue covered by bacterial aggregates in the presence of extracellular material
Control	41±2	0±0
10403S	44±5	11±3^b^
C52	16±2^a^	0±0
EGDe	46±5	62±10^c^
10403SΔ*prfA*	42±4	0±0
EGDeΔ*prfA*	43±5	0±0

Rat ciliated ependymal cells incubated with *L. monocytogenes* strains *in vitro*. At 3 hours post-infection ciliary beat frequency was measured. Data are the mean ± standard deviation of 5 to 11 experiments (taken from a total of 100–220 CBF readings).

a Significantly different from the control (non-infected ependymal cells) p<0.05.

b Significant difference from the C52 (p<0.05) or wild type EGDe (p<0.001) and

c indicates significant difference from 10403S and C52 (P<0.001).

At three hours post-infection there was no significant difference (P>0.05) in the ciliary beat frequency of cells incubated with 10403S and the control (incubated in medium alone; Table 2, Video S2). However, the amplitude of the beating cilia covered by bacterial aggregates was markedly reduced (36% of the control value, P<0.001, Table 3). The ciliary beat amplitude of cilia not covered by bacterial aggregates was also reduced compared to control but to a much lesser extent (88% of the control value, P<0.001, Table 3). To determine if listerial cell free extract effects the cilia, lysates of 10403S (10^8^ cfu) were prepared by the addition of antibiotic (penicillin, ampicillin or gentamicin), these were added to the ependymal cilia, and over 3 hours had no effect on CBF and beat pattern (data not shown).

**Table 3:** Ependymal ciliary beat amplitude inside and outside the listerial aggregates.

*L. monocytogenes* strain	ciliary beat amplitude a outside the listerial aggregates	ciliary beat amplitude a inside the listerial aggregates
Control	100±0	NLA
10403S	88±6^b^	36±4^b^
C52	67±4^b^	NLA
EGDe	81±6^b^	36±3^b^
10403SΔ*prfA*	80±6^b^	NLA
EGDeΔ*prfA*	77±2^b^	NLA

Data are the mean ± standard deviation of 4 to 5 experiments.

a Ciliary beat amplitude is the percentage of the amplitude in the absence of bacteria.

b Significantly different from the control ciliary beat amplitude outside the listerial aggregates. There were no significant differences in the ciliary beat amplitude outside the listerial aggregates in the presence of all listeria strains. NLA: no listerial aggregates.

To investigate if the findings with *L. monocytogenes* strain 10403S were strain specific; the study was repeated with two other wild-type strains of *L. monocytogenes*, C52 and EGDe. After three hours incubation with C52, in contrast to 10403S there was a significant reduction in ciliary beat frequency (P<0.001, Table 2). The bacteria were seen to be attached to and moving in time with the beat of the cilia but no extracellular material or bacterial aggregates were seen. The amplitude of the cilia after incubation with C52 was reduced significantly compared to the control (67% of the control value, P<0.001, Table 3).

**Figure 2:**
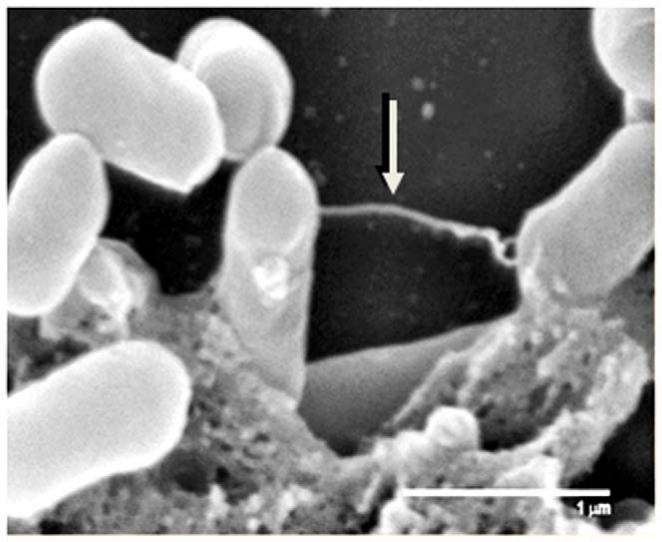
Scanning electron microscope image of listeria and extracellular material. *L. monocytogenes* strain 10403S was embedded within the extracellular material. The arrow is pointing to a strand of extracellular material between the bacteria. Scale bar represents 1 µm.

After three hours incubation with EGDe, there was no decrease in ciliary beat frequency compared to the control (Table 2, Video S1). Incubation with the EGDe strain initially resulted in long chains of bacteria attaching to the cilia, moving at the same beat frequency as the cilia (Video S3). After three hours there were large areas of bacterial aggregates in an extracellular material (Video S4). Approximately 61% of the surface area of the ependymal cell culture was covered by listerial aggregates in association with extracellular material (Table 2, Fig. 1E and 1F). The SEM image in Figure 2 shows strands of material between the wild type EGDe strain. Where aggregates were present, there was a 64% reduction in ciliary amplitude (P<0.001, Table 3). Where cilia were not covered by listerial aggregates, the ciliary beat amplitude was also decreased by 19% (P<0.001, Table 3). The reduction in ciliary amplitude appeared to be dependent on the size of the bacterial aggregates. The ciliary amplitude at 3 hours co-culture was markedly reduced compared to the 2 hour amplitude, at which time the bacterial aggregates were smaller (data not shown). From confocal images we occasionally saw bacteria inside the cells, but the majority of cells were not seen to be infected (data not shown). With each strain, bacterial aggregates and extracellular material formed only during contact between the ependymal cells and the bacteria. No aggregates or extracellular material were visible after incubation of the bacteria and medium taken from the ependymal cell cultures.

### A role for PrfA

Ciliated ependymal cells were incubated with Δ*prf*A mutants of EGDe and 10403S for three hours at 37°C. The C52 Δ*prf*A mutant was not available. In contrast to the wild-types, no aggregates or extracellular material were observed with the Δ*prf*A mutants (Table 2). As with the wild-types, the mutants had no effect on ciliary beat frequency (Table 2). Although there was significant reduction in ciliary beat amplitude of the cells incubated with EGDΔ*prf*A and 10403SΔ*prf*A (P<0.001, Table 3), the reduction was not significantly different (P>0.05) from the wild-types (Table 3).

## Discussion

In this study we investigated the effect of *L. monocytogenes* on ependymal cilia to improve our understanding of the pathophysiology of listerial meningitis. Three aspects of the results are noteworthy: the strain-specific effect of listeria on the ependyma, the presence of aggregates of listeria on the cilia and the dissociation of ciliary beat frequency from beat amplitude.

The introduction of high speed digital video imaging has allowed us to measure the amplitude of beating cilia in an ependymal culture system and to simultaneously determine their ciliary beat frequency [26], [27]. At a concentration seen in the rat brain during listerial meningitis [9], [11], [12], three of the wild-type *L. monocytogenes* strains studied were capable of decreasing the ciliary amplitude. However only strain C52 significantly reduced ciliary beat frequency. A different strain-specific pattern was seen with aggregation around cilia, with EGDe showing the greatest aggregation but no aggregation of C52. Aggregates were most often seen in the presence of extracellular matrix. The reasons for these strain differences are not clear. Inter-strain variation in listeria has been previously reported, for example, in adhesion to abiotic surfaces [28] and in response to sodium chloride and low pH [29]. Those results and the data from this study indicate that caution must be observed when extrapolating from results with a single strain of *L. monocytogenes*.

Adherence of pathogens to host cells is pre-requisite for cell invasion [30], [31]. In this study we showed that listeria attached to ependymal cells but an unexpected observation was the extensive aggregation of the listeria attached to cilia, but no attachment to other parts of the ependymal cells. The rapid adhesion of listeria to ependymal cilia and the marked aggregation of listeria with the formation of extracellular material may help to explain why CSF samples taken clinically from patients with listerial meningitis contain relatively small numbers of listeria. Interaction between the listeria and cilia is necessary for aggregate formation but direct interactions between individual bacteria cells also is occurring. Significantly, aggregates were only found physically attached to the cilia with no aggregates in the culture medium or in spent culture medium. Incubation of each bacterial strain and spent supernatant (supernatant from a previous experiment) did not result in bacterial aggregate formation, indicating that bacterial aggregates and extracellular material formed as a result of contact between the ependymal cells and the bacteria. Although it remains to be determined if this material is secreted by the ependymal cells or the bacteria. It seems that signalling events are occurring, presumably after initial cilium/listeria attachment, resulting in a change in the surface phenotype of the bound listeria, which leads to more bacteria binding. In some instances extracellular material was visible and where present it may contribute to the formation of the aggregate but this material is not a prerequisite for aggregation because aggregates are found in the absence of this material. The formation of these bacterial aggregates may benefit the listeria by stabilising contact with the ependyma and by slowing CSF movement through interference with ciliary functioning, thereby facilitating invasion and decreasing clearance by CSF movement.

Interestingly, we observed a dissociation of ciliary beat frequency from beat amplitude, in that amplitude could be reduced without an effect on frequency. To our knowledge such dissociation has not been reported before. Therefore, in order to determine effects on overall ciliary function, measurement of ciliary beat frequency and pattern should be used. For cilia associated with listerial aggregates, a ‘drag’ effect due to the attached aggregates is likely to contribute to decreased amplitude. However, in the case of C52 no aggregates were formed thus a ‘non-mechanical’ mechanism is possible. It is also possible that the viscosity of the medium surrounding the cilia is increased. However, these points do not explain the maintenance of beat frequency. Indeed, one may expect an increase in frequency if amplitude was reduced. An unchanged frequency of the beat stroke, even when the distance travelled (amplitude) decreases, suggests there is a fixed timing of intra-cilium events between ciliary strokes. Future investigations are required to decipher these mechanisms. However, before we can deduce any pneumococcal mechanism of interference, what can be said is that the successful invasion of ependymal cells by listeria does not appear to play a role in this, because the majority of cells were not infected during the 3 hour time course of this study.

We recently studied the effect of viscosity on ependymal cells [32] and found an increase in viscous loading was followed by a rapid decrease in the frequency of ependymal cilia to a level that was maintained while the increased viscous load was present. The ciliary beat amplitude was not affected with low viscous loads (1–40 cP) but was reduced at higher viscosities (60 cP). These results are similar to the effects of increasing viscosity respiratory and oviductal ciliary beat frequency [33]. Johnson and colleagues [33] found no significant reduction in beat amplitude over a viscosity range of 1–150 cP. Of interest they found that at levels above 180 cP ciliary activity became very unpredictable. Cilia either stopped beating, just slowed down or seemed to beat more quickly with a short amplitude. Insight into the mechanism of autoregulation of ciliary beat frequency following exposure to changes in viscosity was provided by Andrade and colleagues [34]. They found that changes in mucous viscosity activate vanilloid 4-like (TRPV4) channels that elevate intracellular Ca^+2^. They also found channel opening requires the activity of phospholipase A_2_. This occurs at high viscous loads allowing cilia to adapt to a wide range of viscosities.

Bacterial pathogens, including *L. monocytogenes*, are known to form structured populations of microorganisms, adhered and embedded in an extracellular matrix consisting mainly of exopolysaccharides (biofilms) [35]. It is thought that these biofilm structures protect the bacteria from host immune responses, as well as antimicrobials [36]. Bacterial biofilms have been shown to occur in a sequential process generally involving, 1) initial attachment of individual cells to the surface 2) formation of aggregates 3) further cell proliferation and biofilm maturation with polysaccharide formation [37]. It is possible that the aggregates seen in this study are due to the same processes that are important for biofilm formation. The observation that the formation of aggregates and extracellular material was abolished in *ΔprfA* mutant indicates that genes within the regulon of the central virulence regulator, PrfA, are involved. To our knowledge there are no PrfA regulatory genes that have been shown to be involved in aggregate formation. The inhibition of ciliary beat frequency by C52, a strain in which PrfA is in a constitutively activated state [38], [39], also indicates that expression of the PrfA regulon prior to exposure to the ependymal cells affects ciliary functioning. The absolute requirement of PrfA for *L. monocytogenes* virulence has been demonstrated before, as has the requirement for several genes within the PrfA regulon [21], [22], but the explanation of how the regulon determines the effects reported here remains to be determined.

Our future studies will focus on elucidation of the molecular determinants of the events reported.

## Supporting Information

Video S1 Rat brain ciliated ependymal cells after incubation in tissue culture medium 199 for three hours (negative control). A slow motion video of beating cilia on ependymal cells. Note the distance travelled by the tip of each cilium; this is the ciliary amplitude. For all videos the beating cilia were recorded (32x objective) using a troubleshooter 1000 high speed video camera (Lake Image Systems Ltd, UK) at 500 frames per second. Video sequences were played back at a reduced frame rate. For viewing the AVI files, MiDAS 4.0 player software should be used (http://www.xcitex.com/html/downloads.php).
(4.92 MB AVI)

Video S2 Rat brain ciliated ependymal cells after incubation with wild type *L. monocytogenes* strain 10403S for three hours. This slow motion video shows the listerial aggregates attached to and moving at the same frequency as the cilia. Compared to the control, the ciliary amplitude is markedly reduced where associated with the bacterial aggregates.
(8.76 MB AVI)

Video S3 Rat brain ciliated ependymal cells after incubation with wild type *L. monocytogenes* strain EGDe for two hours. This slow motion video shows long chains of the EGDe bacterial strain attached to and moving at the same frequency as the cilia. The bacteria appear to form a network over the underlying cilia. The ciliary beat frequency is normal, however the amplitude of the ciliary beat is reduced compared to the control.
(10.07 MB AVI)

Video S4 Rat brain ciliated ependymal cells after incubation with wild type *L. monocytogenes* strain EGDe for 3 hours. This slow motion video shows large areas of bacteria aggregated to extracellular material and covering the cilia on ependymal cells. The bacteria are moving at the same frequency as the cilia. The ciliary beat frequency is normal but the ciliary beat amplitude is dramatically reduced compared to the control.
(9.07 MB AVI)
